# Segment 2/3 Hypertrophy is Greater When Right Portal Vein Embolisation is Extended to Segment 4 in Patients with Colorectal Liver Metastases: A Retrospective Cohort Study

**DOI:** 10.1007/s00270-018-02159-5

**Published:** 2019-01-17

**Authors:** Christopher J. Hammond, Saadat Ali, Hafizul Haq, Lorna Luo, Judith I. Wyatt, Giles J. Toogood, J. Peter A. Lodge, Jai V. Patel

**Affiliations:** 1 Department of Vascular Radiology, Leeds Teaching Hospitals NHS Trust, Great George Street, Leeds, LS1 3EX UK; 2 University of Leeds Medical School, Leeds, UK; 3 Department of Pathology, Leeds Teaching Hospitals NHS Trust, Leeds, UK; 4 Department of Hepatobiliary Surgery, Leeds Teaching Hospitals NHS Trust, Leeds, UK

**Keywords:** Therapeutic embolisation, Portal vein, Colorectal liver metastasis, Future remnant liver, Hepatectomy, Liver regeneration

## Abstract

**Background:**

In patients with colorectal cancer liver metastases (CRLM), right portal vein embolisation (RPVE) is used to increase the volume of the future remnant liver (FRL) before major hepatic resection. It is not established whether embolisation of segment 4 in addition RPVE (RPVE + 4) induces greater hypertrophy of the FRL. Limitations of prior studies include heterogenous populations and use of hypertrophy metrics sensitive to baseline variables.

**Methods:**

From 2010 to 2015, consecutive patients undergoing RPVE or RPVE + 4 for CRLM, who had not undergone prior major hepatic resection and in whom imaging was available, were included in a retrospective study. Data were extracted from hospital electronic records. Volumetric assessments of segments 2–3 were made on cross-sectional imaging before and after embolisation and corrected for standardised liver volume.

**Results:**

Ninety-nine patients underwent PVE, and 60 met the inclusion criteria. Thirty-eight patients underwent RPVE, and 22 underwent RPVE + 4. Forty-five patients had undergone median 6 cycles of prior chemotherapy. Eighteen patients had FRL metastases at PVE, and 16 had undergone subsegmental metastasectomy in the FRL. Assessments of the degree of hypertrophy (DH) of segments 2/3 were made at median 35 (interquartile range 30–49) days after PVE. RPVE + 4 resulted in a significantly greater increase in DH than RPVE (7.7 ± 1.8% vs 11.3 ± 2.6%, *p* = 0.011). No confounding association between baseline variables and the decision to undertake RPVE or RPVE + 4 was identified. Median survival was 2.4 years and was not influenced by segment 4 embolisation.

**Conclusion:**

RPVE + 4 results in greater DH of segments 2/3 than RPVE in people with CLRM.

## Introduction

An increase in volume of non-embolised segments of liver after embolisation of segmental branches of the portal vein was initially described in the mid-1980s [1]. Patients in whom major liver resection is planned are at risk of post-operative liver insufficiency where the portion of liver to be left in situ [the future remnant liver (FRL)] is small. The minimum limit of the FRL is 20–40% of total liver volume dependent on the presence of background liver disease [2–5]. In patients in whom the FRL will be small, portal vein embolisation (PVE) increases FRL volume [6–8] and function [9–11].

For patients with disease in segment 4, removal of this segment with the right lobe (extended right hemihepatectomy) may be required. Extended right hemihepatectomy typically leaves only segments 2 and 3 in situ [12], and these segments frequently account for less than 20% of the total liver volume [13]. Tumours in non-embolised segments of liver may progress more rapidly after PVE [14, 15], of importance for disease in segment 4 given its proximity to the resection margin. For these reasons, embolising all of the liver to be resected (segments 4–8) and maximising the hypertrophic stimulus to segments 2 and 3 may be appropriate. Extension of PVE to segment 4 may result in technically easier extended right hemihepatectomy [16].

Embolisation of the segment 4 portal vein(s) is technically challenging [16, 17], risks non-target embolisation to the FRL [18, 19] and may be associated with increased complication [20, 21]. Some authors have found no additional hypertrophy with right portal vein and segment 4 embolisation [RPVE + 4] compared with embolisation of the right lobe alone (RPVE) [3, 18, 22, 23].

For these reasons, there has been debate about whether RPVE + 4 is worthwhile. Comparisons between studies are difficult due to variations in incidence of underlying liver disease, indications for resection (metastatic disease or primary liver cancers), prior chemotherapy exposure, the embolic agent used, the initial size of the FRL and the volume metrics used to assess outcome.

We wished to determine whether hypertrophy of segments 2 and 3 is influenced by segment 4 embolisation in a homogenous population of patients with colorectal liver metastases (CRLM) and whether the decision to undertake segment 4 embolisation is associated with differences in survival.

## Methods

Our institution’s radiology results server was interrogated for patients undergoing PVE between 1 January 2010 and 31 December 2015. Demographic, diagnostic, biochemical and procedural data were abstracted from the medical records of these patients.

Patients undergoing PVE for diagnoses other than CRLM and patients with CRLM who had undergone prior major liver resection [defined as resection of one or more segments or associated liver partition and portal vein ligation for staged hepatectomy (ALPPS) procedures] were excluded. The immediate pre-PVE imaging and immediate pre-hepatic resection imaging of eligible patients were reviewed. Assessment of underlying liver pathology (including fibrosis and chemotherapy associated liver damage) was made by histological review of resected liver tissue.

Volumes of segments 2/3 were estimated by manually tracing around the edge of these segments on regularly spaced 1-, 3- or 5-mm-thick axial slices, summing the areas and multiplying by the interval between measured slices. The majority of intervals between measured slices was 10 mm, and no interval was more than 12 mm. The anatomical landmark used for the lateral aspect of segments 2/3 was the vertical oblique plane containing the falciform ligament and left hepatic vein.

All PVE procedures were undertaken via an ultrasound-guided percutaneous puncture into a portal vein radicle. RPVE + 4 was undertaken where metastatic disease in segment 4 was not amenable to metastasectomy and extended right hemihepatectomy was planned. Where there were segment 4 metastases amenable to metastasectomy (or no segment 4 metastases), RPVE and right hemihepatectomy were planned.

For survival analysis, patients were censored by the date of their last interaction with the hospital assessed at 31.12.2017.

The degree of hypertrophy (DH) of segments 2/3 was calculated by subtracting the volume of these segments before embolisation from their volume on the last scan before major hepatic resection (*V*_post_− *V*_pre_) and correcting for standardised liver volume (sLV). sLV is derived as a function of body surface area (BSA).

$${\text{DH}} = \frac{{\left( {V_{{\text{post}}} - V_{{\text{pre}}} } \right)}}{\text{sLV}}$$

where $${\text{sLV}} = \left( {1267.{\text{BSA}}} \right) - 794$$ [24].

BSA was determined using the Mosteller method [24].

### Statistical Analysis

Results for normally distributed continuous variables are presented as mean ± SD with comparisons made with a *t* test and Pearson’s correlation coefficient. Comparisons between other continuous variables were with a between-groups Mann–Whitney test. Categorical variables were compared with Fisher’s exact test. Durations are presented as median and interquartile ranges (iqr). Survival was assessed using Kaplan–Meier analysis, with the log-rank test used to compare groups.

## Results

Between 1 January 2010 and 31 December 2015, 99 patients underwent PVE. Of these, 26 were excluded for alternative diagnoses (hepatocellular carcinoma—15; cholangiocarcinoma—11) and nine were excluded as they had had undergone prior major hepatic surgery. Two patients were lost to follow-up.

**Fig. 1:**
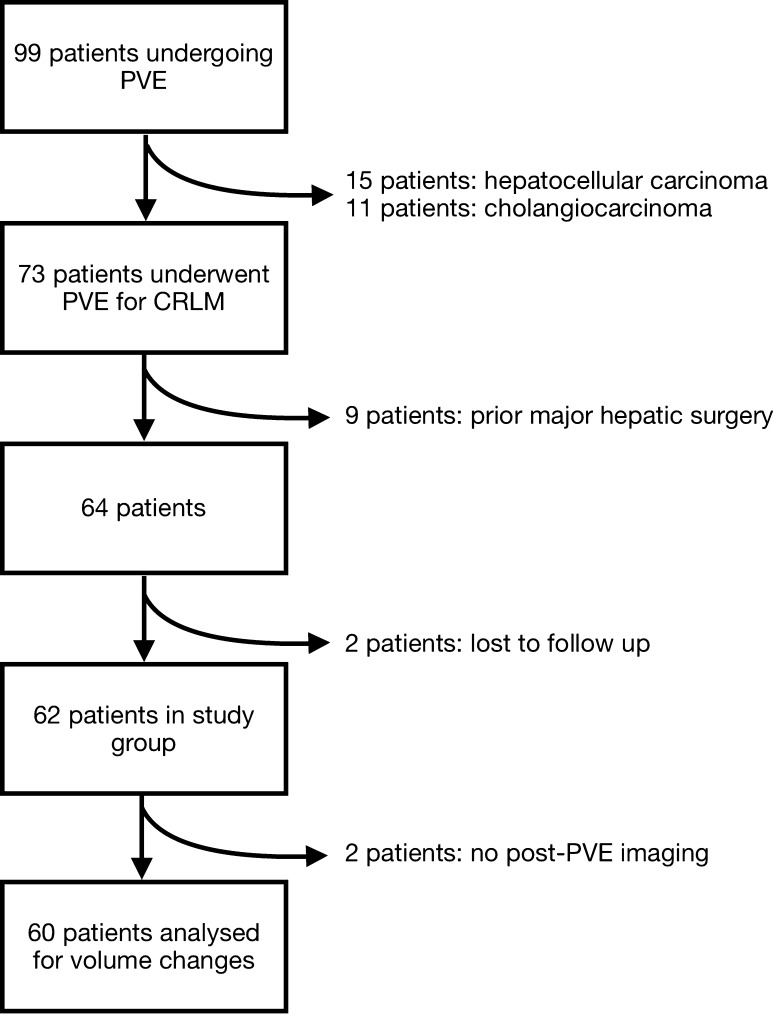
CONSORT diagram of the selection process for inclusion in the study from an initial cohort of 99 patients coded as undergoing PVE between 1 January 2010 and 31 December 2015

Of the remaining 62 patients, two patients had no post-PVE imaging (one from each group): one patient’s imaging could not be retrieved from PACS, and one patient died before any post-procedural imaging could be undertaken. These patients were included in mortality analyses but not in volume analyses. Sixty patients were therefore assessed for segment 2/3 hypertrophy (Fig. 1).

**Fig. 2:**
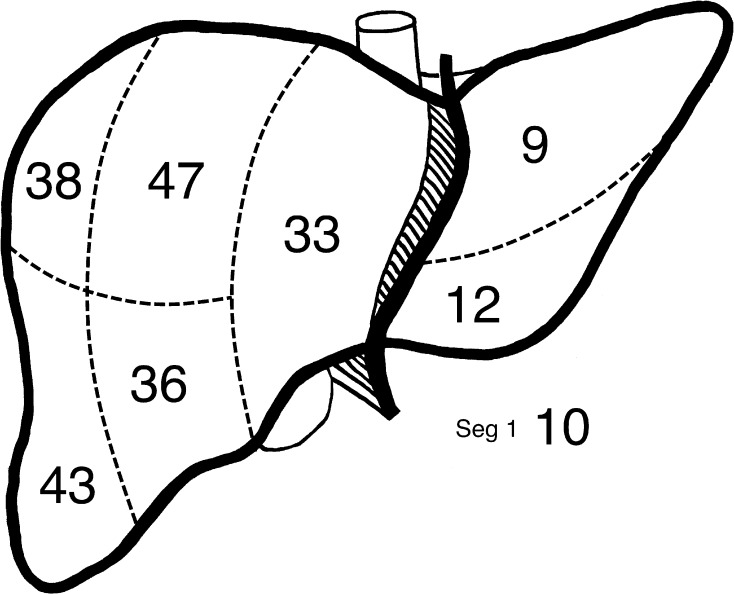
Location and number of metastases in the 62 included patients undergoing PVE

Median age at PVE was 69.8 years. Thirty-nine patients were male, and 11 patients were diabetic (Table 1). The distribution of metastases pre-PVE is illustrated in Fig. 2.

**Table 1:** Influence of potential confounding variables on the cohorts of patients undergoing RPVE and RPVE + 4

	RPVE (*n* = 38)	RPVE + 4 (*n* = 22)	*p*-Value
Age (years)	70.2 (iqr 63.8–75.7)	66.7 (61.1–74.6)	0.42^‡^
Sex			
Male	27	12	0.26*
Female	11	10	
Diabetes			
No	32	17	0.10*
Yes	6	5	
Segment 2/3 metastases at PVE			
No	26	18	0.37*
Yes	12	4	
Prior segment 2/3 metastasectomy			
No	28	18	0.54*
Yes	10	4	
Prior chemotherapy			
No	11	4	0.54*
Yes	27	18	
Prior treatment with irinotecan			
No	36	20	0.62*
Yes	2	2	
Prior treatment with bevacizumab			
No	36	22	0.53*
Yes	2	0	
Number of cycles of chemotherapy (45 patients undergoing chemotherapy)	4(iqr 0–6)	5(iqr 3–6)	0.56^‡^
Non-target embolisation			
No	34	17	0.27*
Yes	4	5	
SOS present (42 patients with pathology available)			
No	27	12	1.00*
Yes	2	1	
Steatosis score ^§^(42 patients with pathology available)	0(iqr 0–0)	0(iqr 0–1)	0.19^‡^
Pre-embolisation segment 2/3 volume as proportion of sLV (= *V*_pre_/sLV)	18.9%	19.2%	0.40^‡^
	(iqr 10.9–23.5)	(iqr 15.4–22.9)	
Resection margins (46 patients undergoing resection)			
R0	20	7	0.34*
R1	11	8	
Days PVE to CT assessment	39	32	0.09^‡^
	(iqr 31–49)	(iqr 23–49)	

Summary statistics are: number or median (interquartile range)

*Fisher’s exact test

^‡^Mann–Whitney test

^§^0: < 5%; 1: 5-30%; 2: > 30%

Forty-five patients had undergone median 6 cycles of adjuvant or neo-adjuvant chemotherapy with oxaliplatin-based regimens (FOLFOX or OxCap). Six patients had received additional irinotecan or bevacizumab chemotherapy (Table 1). Fourteen patients had undergone prior segment 2/3 metastasectomy at median 37 (iqr 21–143) days before PVE.

No patient had ascites, encephalopathy or stigmata of portal hypertension. Pathological specimens were available in 42 of 46 patients undergoing liver resection. Twenty patients had no steatosis, nine had less than 2% steatosis, 11 had 5–30% steatosis and two had 30–60% steatosis. There was evidence of steatohepatitis in three patients with bridging fibrosis in one. Another patient demonstrated fibrosis. Three patients had pathological changes of sinusoidal obstructive syndrome [SOS].

Two patients (one each from RPVE and RPVE + 4 groups) had incomplete right-sided embolisation due to operator error, resulting in three segments (rather than four) being embolised. Twenty-three patients underwent RPVE + 4, and 39 patients underwent RPVE. Excluding the two patients without post-PVE imaging, there were 22 patients in the RPVE + 4 group and 38 in the RPVE group.

Two patients in the RPVE group had difficult right-sided access and underwent left lobe puncture and right portal vein embolisation with coils and Gelfoam (Pfizer, New York, USA). In all other patients, a right-sided puncture was undertaken, and the right portal veins were embolised with Glubran cyanoacrylate glue (GEM, Viareggio, Italy) diluted 1:3 (median) in lipiodol. In patients undergoing RPVE + 4, segments 4 were embolised with Glubran (14 patients), coils (12 patients), particulate agents (three patients) or Amplatzer plugs (St. Jude Medical, Minnesota, USA, two patients) or combinations of these agents.

Minor (non-flow limiting) non-target embolisation to small peripheral third- or fourth-order branches of the FRL was seen in nine patients, eight with glue and one with a coil. The coil was retrieved. A single patient in the RPVE + 4 group developed a large sub-capsular haematoma post-PVE, managed conservatively. The patient died suddenly 22 days post-PVE from pulmonary embolism. There was no other significant peri-procedural mortality or morbidity.

Follow-up CT imaging was undertaken at median 35 days post-PVE (iqr 30–49 days). Three patients had very slow growth with satisfactory hypertrophy only evident on repeat imaging over 100 days post-PVE despite a technically complete and uncomplicated procedure.

Initial segment 2/3 volume (*V*_pre_) and proportion of sLV was 280.9 ± 38.4 ml versus 310.3 ± 46.0 ml (no difference, two-tailed *t*-test *p* = 0.33) and 17.5 ± 2.6% vs 19.1 ± 2.7 (no difference, two-tailed *t*-test *p* = 0.41) in the RPVE and RPVE + 4 groups, respectively.

**Fig. 3:**
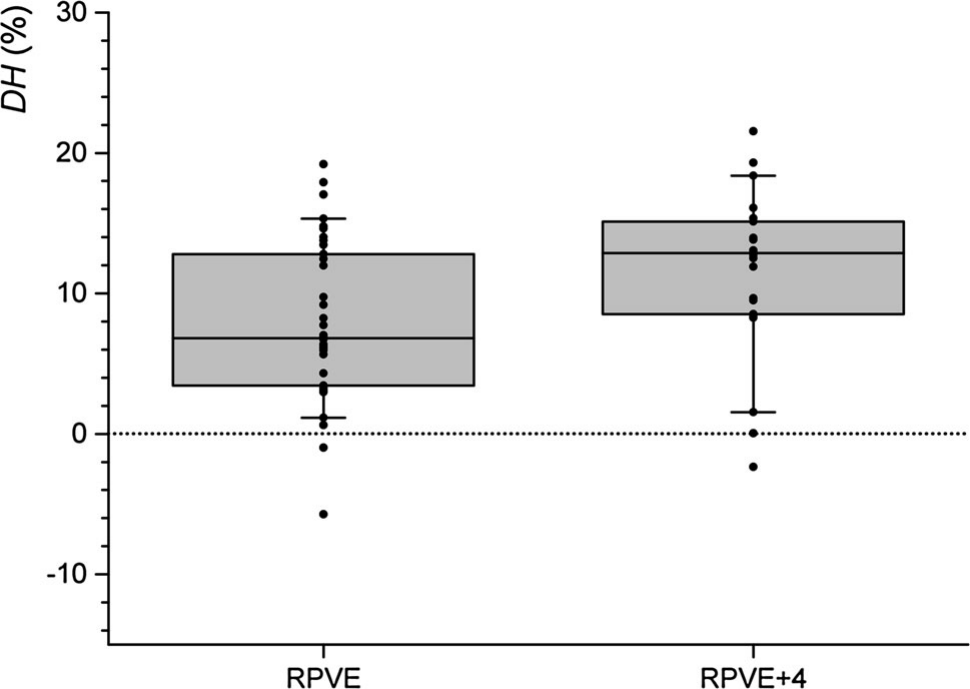
Box and whisker plot of DH in the RPVE and RPVE + 4 groups. The upper and lower bounds of the box represent the upper and lower quartiles of the cohort, respectively. The central line across the box is the median value. The whiskers extend to the 90th and 10th centile. Individual values of DH are plotted as points. For simplicity, overlapping values of DH are plotted as a single point

DH was normally distributed. DH was significantly greater in the RPVE + 4 group than in the RPVE group (11.3 ± 2.6% vs 7.7 ± 1.8%; single tailed *t*-test, *p* = 0.011), Fig. 3. This result was insensitive to excluding patients with left lobe non-target embolisation, incomplete right lobe embolisation or the three outlying patients with very slow growth.

For patients undergoing RPVE + 4, segment 2/3 represents the whole of the FRL. In these patients, there was a significant negative correlation between DH and pre-embolisation segment 2/3 volume as a proportion of sLV (i.e. greater hypertrophy in smaller initial segments 2/3. Pearson correlation *p* = 0.006).

In 13 patients, no surgery was attempted due to early post-PVE death (one patient, RPVE + 4) or disease progression (12 patients, six each group, no difference, *p* = 0.33). Median interval from PVE to surgery was 78 days (iqr 68–106 days). Surgery was abandoned after laparotomy in three patients due to the presence of unexpectedly extensive disease. Forty-five patients underwent the liver resection that had been planned, and one patient underwent multiple metastasectomies (the liver was considered too fatty to allow major resection).

A single patient died within 90 days of surgery (day 78). Three patients developed clinical evidence of liver insufficiency with ascites and jaundice, all managed conservatively.

**Fig. 4:**
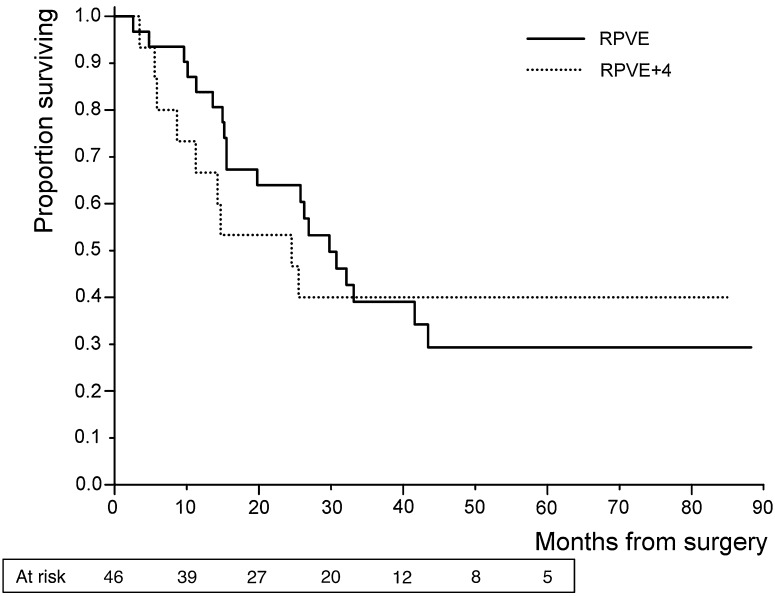
Kaplan–Meier survival analysis for 46 patients undergoing liver surgery by embolisation performed (RPVE vs RPVE + 4)

For patients who underwent liver surgery, median survival after surgery was 26.0 months and 3-year survival was 39%. There was no difference in survival for patients who underwent liver resection between the RPVE and RPVE + 4 groups (Fig. 4, log-rank comparison *p* = 0.83). There was no difference in survival after PVE between the RPVE and the RPVE + 4 groups (log rank *p* = 0.39). Median survival after PVE was 24.3 months.

## Discussion

### Choice of Metrics of Hypertrophy

There is no uniformly accepted metric of the fractional contribution of the FRL to whole liver volume or of hypertrophy following PVE [6]. The use of total (non-tumour) liver volume (TLV) pre- and post-PVE may be associated with substantial cumulative error [24] and does not account for right lobe atrophy after PVE [6, 18]. Metrics of hypertrophy describing changes in FRL volume *relative* to baseline are sensitive to the initial size of the FRL (*V*_pre_). DH is unaffected by variations in patient size, *V*_pre_, atrophy of embolised segments or size and number of metastases and is a widely used metric of FRL hypertrophy [3, 7, 17, 20, 23, 25–28].

### Volumetric Analysis

We identified a statistically significant increase in segment 2/3 hypertrophy when RPVE + 4 is compared with RPVE. This is consistent with the findings of a multivariate regression analysis of 360 patients undergoing PVE at a single institution in the USA [26], other publications on iterations of this data set [20, 21] and the findings of other researchers [19]. These publications include patients with various indications for planned liver resection and incidences of underlying liver disease or report metrics sensitive to patient size [21].

In contrast to our findings, other investigators [3, 18, 22, 23] were not able to differentiate FRL hypertrophy with RPVE + 4 from that observed with RPVE. Of these studies, only Ribero et al. [3] and Massimono et al. [23] reported DH, the other studies reporting metrics either sensitive to right lobe atrophy [18] or *V*_pre_ [22].

Background liver disease is known to affect hypertrophy in response to PVE [6, 29, 30], and the negative findings of Ribero et al. [3] and Capussotti et al. [22] may be due to the incidence of chronic liver disease (present in over a quarter of patients) in their cohorts. In contrast, only three patients in our cohort had steatohepatitis and only two had bridging fibrosis. In a small cohort of patients most of whom had no evidence of underlying liver pathology, Massimino et al. [23] could not demonstrate additional FRL hypertrophy with RPVE + 4. They ascribed this to lack of statistical power as there was a trend to greater hypertrophy in the RPVE + 4 group.

Pre-embolisation FRL size is an important correlate of DH [18, 31, 32] (a smaller FRL being associated with a greater degree of hypertrophy) and may account for most (if not all) of the additional hypertrophy observed with RPVE + 4 (where the FRL is 1 segment smaller) [18]. The present study confirms this finding. In the studies by Capussotti et al. [22] and de Baere et al. [18], pre-embolisation FRL volume in the RPVE and RPVE + 4 groups was similar. It is therefore not unexpected that these investigators could not differentiate FRL hypertrophy between RPVE and RPVE + 4 as the volumetric stimulus will also have been similar. We found no confounding association between initial segment 2/3 volume and the decision to undertake RPVE or RPVE + 4 (Table 1), and therefore our results cannot be explained on the basis of smaller segment 2/3 volume in the RPVE + 4 group.

### Potential Confounders

We found no confounding association between any baseline variables and the decision to undertake RPVE or RPVE + 4 (Table 1). In particular, there was no difference in prior exposure to chemotherapy [26, 27, 33], prior segment 2/3 metastasectomy [18, 19, 21], time from PVE to imaging or presence of segment 2/3 metastatic disease at PVE between the two groups (Table 1). We based our analysis on intention to treat irrespective of whether the PVE was technically successful. However, excluding patients with incomplete right lobe embolisation or left lobe non-target embolisation did not affect our result.

### Complications and Survival

Our rates of disease progression to non-resectability (21%) and significant complication after PVE (1.6%) are within published limits [6, 7, 34] and European quality improvement guidelines [35]. Our cohort’s overall 3-year survival is consistent with prior reports of survival after PVE and hepatic resection [36–38]. Our cohort of patients is therefore not unusual in either disease extent or outcome, and therefore our results are reasonably generalisable.

We found no survival detriment for patients undergoing RPVE + 4 overall or for those undergoing liver resection, i.e. more extensive embolisation is not associated with increased hazard of death. Given the relatively low numbers of patients undergoing surgery in our study, this finding must be interpreted with caution, but appears consistent with a recent meta-analysis of outcome after major hepatectomy with or without prior PVE [39].

### Limitations

The present study was retrospective and therefore is limited by the biases inherent to this type of investigation. In particular, we cannot exclude the possibility that our results are due to unknown patient factors associated with the decision to offer extended right hemihepatectomy and RPVE + 4.

## Conclusion

Our data suggest that in patients with CRLM, RPVE + 4 is associated with increased hypertrophy of segments 2 and 3 compared with RPVE. We recommend that all patients with CRLM in whom extended right hemihepatectomy is planned and in whom the FRL is small (< 20% of sLV) undergo RPVE + 4.
